# Larval midgut modifications associated with *Bti* resistance in the yellow fever mosquito using proteomic and transcriptomic approaches

**DOI:** 10.1186/1471-2164-13-248

**Published:** 2012-06-15

**Authors:** Guillaume Tetreau, Krishnareddy Bayyareddy, Christopher M Jones, Renaud Stalinski, Muhammad A Riaz, Margot Paris, Jean-Philippe David, Michael J Adang, Laurence Després

**Affiliations:** 1 Laboratoire d’Ecologie Alpine, LECA-UMR 5553, Université de Grenoble 1, BP 53, 38041, Grenoble cedex 09, France; 2 Department of Entomology, University of Georgia, Athens, GA, 30602-2603, USA; 3 Vector Group, Liverpool School of Tropical Medicine, Liverpool, L3 5QA, UK; 4 Department of Biochemistry and Molecular Biology, University of Georgia, Athens, GA, 30602-2603, USA

**Keywords:** *Aedes aegypti*, *Bacillus thuringiensis israelensis*, DIGE, Microarray, RT-qPCR, Resistance, Transcriptomics, Proteomics, Midgut, Mosquito, Candidate genes

## Abstract

**Background:**

*Bacillus thuringiensis* var. *israelensis* (*Bti*) is a natural larval mosquito pathogen producing pore-forming toxins targeting the midgut of Diptera larvae. It is used worldwide for mosquito control. Resistance mechanisms of an *Aedes aegypti* laboratory strain selected for 30 generations with field-collected leaf litter containing *Bti* toxins were investigated in larval midguts at two levels: 1. gene transcription using DNA microarray and RT-qPCR and 2. differential expression of brush border membrane proteins using DIGE (Differential In Gel Electrophoresis).

**Results:**

Several *Bti* Cry toxin receptors including alkaline phosphatases and N-aminopeptidases and toxin-binding V-ATPases exhibited altered expression levels in the resistant strain. The under-expression of putative *Bti-*receptors is consistent with *Bt*-resistance mechanisms previously described in Lepidoptera. Four soluble metalloproteinases were found under-transcribed together with a drastic decrease of metalloproteinases activity in the resistant strain, suggesting a role in resistance by decreasing the amount of activated Cry toxins in the larval midgut.

**Conclusions:**

By combining transcriptomic and proteomic approaches, we detected expression changes at nearly each step of the ingestion-to-infection process, providing a short list of genes and proteins potentially involved in *Bti*-resistance whose implication needs to be validated. Collectively, these results open the way to further functional analyses to better characterize *Bti-*resistance mechanisms in mosquitoes.

## Background

Mosquito control represents a major public health concern as mosquitoes transmit many pathogens causing fatal human diseases including malaria, filariasis, dengue, yellow fever, and Chikungunya [1]. Vector borne diseases represent a major health threat and economic burden in disease-endemic countries and are currently expanding worldwide [2,3]. As no specific treatment exists for most of these diseases, the most effective way of reducing the incidence of these diseases is to control the vector mosquitoes [4,5]. Chemical insecticides still used in endemic countries have shown their limits as resistance has evolved in all target species together with environmental concerns due to their high persistence and toxicity for non-target organisms, including humans [6].

The bacterium *Bacillus thuringiensis* var. *israelensis* (*Bti*) produces a mosquitocidal toxic crystal during sporulation and represents the best alternative to chemical insecticides for mosquito larval control due to its high potency and specificity [7]. The action of *Bti* begins when larvae ingest *Bti* spores and toxic crystals. In susceptible larvae, the toxic crystal is dissolved in the alkaline pH of the midgut, protoxins are then activated by digestive proteases to activated-toxins that bind to specific membrane receptors, form pores, disrupt the midgut epithelium, allowing spore penetration and bacterial proliferation in the host tissues [7,8]. The receptors for mosquitocidal *Bti* Cry toxins are similar to the lepidopteran-active Cry toxins which utilize N-aminopeptidase, alkaline phosphatase and cadherin proteins as midgut receptors [9].

In contrast to *Bacillus thuringiensis* subspecies active against lepidopteran and coleopteran species where cases of insect resistance in the field have been reported [10-13], only one study reported *Bti* resistance in field mosquitoes [14]. However, subsequent confirmations of this case have not been reported. The delay in the evolution of resistance to *Bti* is believed to be due to its composite toxic crystal containing four major toxins (Cry4Aa, Cry4Ba, Cry11Aa and Cyt1Aa) [7]. Cyt toxins are known to largely enhance Cry toxins activity due to synergic effects and to drastically decrease resistance development [8,15]. Although *Bti* is known to have a low persistence in the environment, recent studies suggest that it can persist and possibly proliferate in specific conditions [16-18]. In the French Rhône-Alpes region, decaying leaf litters collected in mosquito breeding sites several months after a *Bti* treatment revealed a high toxicity against mosquito larvae due to the presence of large amounts of *Bti*[16]. This toxic leaf litter was used to select an *Aedes aegypti* strain in laboratory conditions. After 18 generations, the selected strain (named LiTOX) was only moderately resistant to the whole *Bti* toxins mixture, but up to 30 fold resistant to individual Cry toxins [19]. Although resistance to *Bti* has already been selected in laboratory conditions [20,21], this is the only reported case of resistance obtained by using field-collected material containing residual *Bti* toxins. Therefore, this *Bti*-resistant LiTOX strain provides a unique opportunity to better understand the mode of action of *Bti* toxins and to elucidate the mechanisms of resistance developed by mosquitoes exposed to field residual *Bti* toxins.

To identify the resistance mechanisms developed by the LiTOX strain, a genome scan and a transcriptome scan were previously performed on whole larvae twelve generations ago [22,23]. The main bias of these whole-larvae approaches is that many genes are identified that may not be directly related to *Bti* resistance. Indeed, selection was shown to have induced many changes in the LiTOX strain, including decreased egg survival to desiccation, longer larval development time and decreased female fecundity [24], reflecting the evolution of resistance costs that are not directly involved in resistance to *Bti* toxins. Because insect midgut is the primary target site for *Bti* toxins our aim in the present work is to focus on constitutive expression changes in midgut proteins of resistant versus susceptible larvae. For that purpose, we combine a comparative analysis of brush border membrane proteins using 2D-DIGE (2-Dimensional Differential in Gel Electrophoresis) with a midgut transcriptome profiling using DNA microarrays. In addition, altered gene expression of known *Bti* Cry toxins receptors (i.e. alkaline phosphatases, cadherins, N-aminopeptidases) between the two strains were investigated using RT-qPCR. Finally, because the DiGE didn’t allow detecting proteins with high molecular size such as cadherins, we performed Western blots with anti-cadherins antibodies.

## Results

### Resistance levels to *Bti* toxins in the LiTOX strain

After 30 generations of selection with leaf litter containing *Bti*, bioassays indicated that the LiTOX strain exhibited a moderate 3.5-fold resistance to commercial *Bti* mixture Vectobac® WG compared to the susceptible strain at the larval stage (Table 1). When *Bti* Cry toxins were tested separately, the LiTOX strain showed an increased resistance of 68-fold, 9-fold and 9-fold to Cry4Aa, Cry4Ba and Cry11Aa protoxins respectively. The relatively important variability observed for the LC_50_ for Cry4Aa toxin of the LiTOX strain is mainly due to a higher variability in larval mortality in the replicates than for the susceptible strain and for the other toxins. As resistance is not fixed yet in the LiTOX strain [24], this variability between replicates might reflect a large range of different combinations of Cry4A resistance alleles between individuals. 

**Table 1:** **Lethal concentrations and resistance ratio for the LiTOX and susceptible strains for*Bti*and Cry toxins**

**Toxins**	**Strain**	**LC**_**50**_**in ng/mL (95% CI)**	**RR**_**50**_
Cry4Aa	Susceptible	646.28 (514.20–826.93)	/
	LiTOX	43873.12 (28396.11–78207.31)	**67.9 fold**
Cry4Ba	Susceptible	322.27 (228.39–468.57)	**/**
	LiTOX	2922.26 (1924.95–4168.76)	**9.1 fold**
Cry11Aa	Susceptible	156.14 (112.86–219.46)	**/**
	LiTOX	1434.81 (1146.52–1774.22)	**9.2 fold**
*Bti* Vectobac WG	Susceptible	90.6 (79.89–101.12)	**/**
	LiTOX	312.6 (277.27–359.49)	**3.5 fold**

**Lethal concentrations 50% (LC**_**50**_**) of the resistant (LiTOX) and the susceptible strain for the three Cry toxins (Cry4Aa, Cry4Ba and Cry11Aa) and for the commercial*Bti*at 24 h. Resistance ratios 50 (RR**_**50**_**) are calculated for each product as LC**_**50**_**of LiTOX divided by LC**_**50**_**of Bora- Bora strain. LC**_**50**_**are expressed in ng/mL.**

### Midgut transcriptome profiling

**Figure 1:**
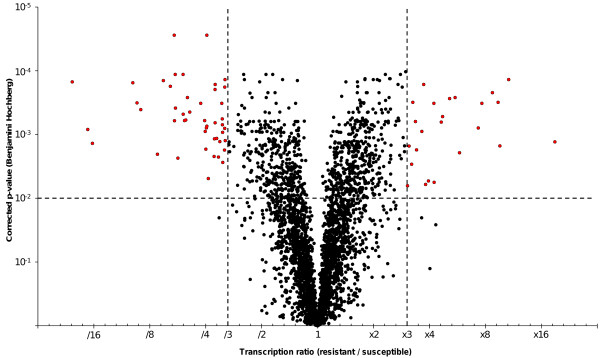
**Volcano plot of differentially-transcribed genes identified by microarray analysis.** The Benjamini-Hochberg P-values were plotted against the fold change in gene expression for all genes. The horizontal lines in the plot represent the statistical test significance 0.01 and the vertical bars represent the genes at least three-fold up- or down-regulated in LiTOX *Bti-*resistant strain compared to Bora-Bora susceptible strain.

Comparative transcriptome profiling between total mRNAs extracted from midguts of larvae from the LiTOX and the susceptible strains was performed using a DNA microarray representing 14204 of the more than 17000 *Ae. aegypti* transcripts identified in Vectorbase. A total of 3512 transcripts were detected in at least 5 hybridizations out of 6 [ArrayExpress: E-MTAB-1094] (Additional file 1). Among them, 24 and 46 genes were significantly over- and under-transcribed respectively in the LiTOX strain (≥3-fold and corrected P-value <0.01) (Additional file 2). Distribution of transcription ratios was well balanced between over and under-transcription ranging from 20.9-fold under-expression to 18.9-fold over-expression (Figure 1). RT-qPCR validation of transcription ratios for 15 selected genes revealed a good correspondence between the two techniques, supporting the reliability of microarray data (Additional file 3).

**Figure 2:**
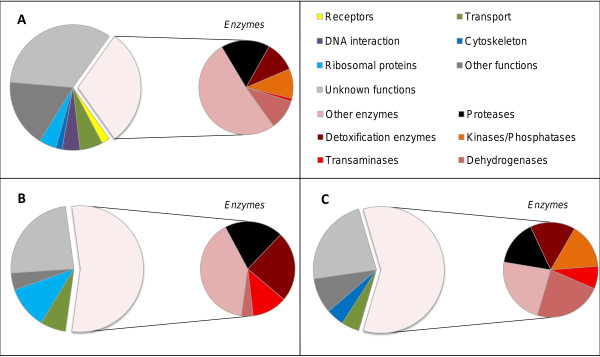
**Functional analyses of all the genes detected and genes differentially expressed in the resistant strain.** Circle charts of the biological functions of all the genes detected (**A**), those under-expressed (**B**) and over-expressed (**C**) in the LiTOX strain compared to the susceptible Bora-Bora strain. Genes are classified into 13 categories: receptors (orange), transport (green), DNA interaction (purple), cytoskeleton (dark blue), ribosomal proteins (light blue), proteases (black), detoxication enzymes (brown), kinases/phosphatases (orange), transaminases (red), dehydrogenases (dark pink), other enzymes (pink), other functions (dark grey) and unknown functions (light grey).

Differentially transcribed genes were further analyzed according to their biological function by classifying them into 13 different categories (Figure 2). Genes of unknown function represented 34% of detected transcripts while genes not assigned to any category (other functions) represented 17%. Enzymes represented 30% of detected transcripts and were strongly over-represented among under- and over-transcribed genes (55% and 60% respectively) (Figure 2B & C). Proteases were equally represented in over- and under-expressed genes, while detoxification enzymes were more often under- than over-transcribed (7 under-transcribed versus 2 over-transcribed genes). Transaminases, represented by only 11 genes in the *Ae. aegypti* genome, were over-represented in under- and over-transcribed genes while dehydrogenases were strongly over-represented only in over-transcribed genes (23% of enzymes compared to 10% overall).

### Midgut differential proteomics

**Figure 3:**
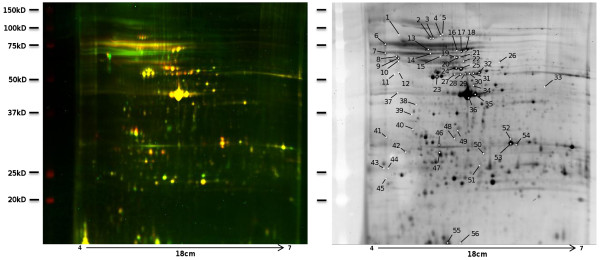
**2D-DIGE gel and corresponding picked silver stained gel.** BBMV proteins were prepared from resistant and susceptible *Aedes aegypti* larval midguts and separated using 2D-DIGE. The spots appear in yellow when corresponding to proteins present at approximately equal amounts in both resistant and susceptible BBMV samples, green for those only present in the susceptible BBMV labeled with Cy3, and red for those only present in the resistant BBMV labeled with Cy5. The x-axis shows pI values from 4 to 7 and the y-axis shows apparent molecular weight in kilodaltons (kDa). Panel A. Overlay of Cy3 and Cy5, and Panel B. Gel co-run stained with deep purple. All the 56 spots picked either on the first, the second or both the two gels, corresponding to the two biological replicates, are noted on this gel.

Midgut membrane proteins were compared between larvae of the LiTOX and susceptible strains using 2D-DIGE (Figure 3). Dye-swapping for each biological sample showed no dye-dependent spot changes on the gels (Additional file 4). Spot locations were reproducible between the biological replicates, but the signal intensity was higher for the second replicate, revealing additional spots differing between the two strains (Additional file 4). A total of 56 distinct protein spots differently expressed between the two strains were processed and 35 unique proteins were identified (Figure 3B, Additional file 5). The MS/MS analyses gave the same protein identifications between biological replicates for spots 2, 8, 20, 21 and 24 with high Mascot scores (from 110 to 249) while spots 14, 42 and 49, showing Mascot scores lower than 100, were assigned to different proteins (Additional file 5). Indeed, none of the spots with low Mascot scores were considered for further analyses. Different spots yielded the same identified protein for 10 proteins, with a maximum of six spots for AAEL005798 (V-ATP synthase subunit beta).

### Genes and proteins differentially expressed in the LiTOX strain

Proteome analysis identified two N-aminopeptidase proteins (APN, annotated as ‘protease m1 zinc metalloprotease’) differentially expressed in the LiTOX strain (Table 2): two spots matching APN AAEL012774 were up-regulated and two of the three spots matching APN AAEL012776 were down-regulated in the LiTOX strain. Transcriptomic approach detected thirteen APN (including AAEL012774 and AAEL012776) with transcription level ranging from −1.82 to +1.96 fold changes (Additional file 1) but none was significant.

**Table 2:** Protein identification of 30 spots with highest Mascot scores picked on deep purple stained 2D-gel

**Spot Nb**	**Fold changes**		**Vectorbase access number**	**Mascot score**	**Top ranking match**	**Predicted PI**	**Predicted mass (kDa)**	**% sequence coverage**	**Species**
*Proteases*									
2	−	1.88	AAEL015386_a	249	dipeptidyl-peptidase	4.91	84.9	42	*Ae. aegypti*
3	−	2.77	AAEL015386_b	149	dipeptidyl-peptidase	4.91	84.9	22	*Ae. aegypti*
4	+	1.81	AAEL012774_a	237	protease m1 zinc metalloprotease	4.81	102.5	41	*Ae. aegypti*
5	+	2.34	AAEL012774_b	162	protease m1 zinc metalloprotease	4.81	86.7	42	*Ae. aegypti*
20	+	1.81	AAEL012776_a	135	protease m1 zinc metalloprotease	5.19	103.3	30	*Ae. aegypti*
21	−	2.74	AAEL012776_b	146	protease m1 zinc metalloprotease	5.19	103.3	27	*Ae. aegypti*
19	−	3.81	AAEL012776_c	136	protease m1 zinc metalloprotease	5.19	103.3	22	*Ae. aegypti*
*Detoxification enzymes*									
45	+	3.01	CPIJ019700	119	cytochrome P450	7.6	58.4	41	*C. quinquefasciatus*
*Kinases-Phosphatases*									
22	−	1.58	AAEL003313	62	alkaline phosphatase	5.46	61.0	23	*Ae. aegypti*
24	−	1.92	AAEL003298_a	186	alkaline phosphatase	5.28	58.8	39	*Ae. aegypti*
25	−	1.16	AAEL003298_b	194	alkaline phosphatase	5.23	58.3	39	*Ae. aegypti*
*Other enzymes*									
6	+	1.78	AAEL010532	146	alpha-amylase	4.82	68.9	37	*Ae. aegypti*
13	−	1.27	AAEL004580	129	beta-galactosidase	4.87	74.1	34	*Ae. aegypti*
11	−	2.06	AAEL002827_a	61	ATP synthase beta subunit	5.03	53.9	32	*Ae. aegypti*
23	−	2.29	AAEL002827_b	152	ATP synthase beta subunit	5.03	53.9	55	*Ae. aegypti*
16	−	2.06	AAEL008787_a	162	V-ATP synthase subunit alpha	5.26	68.5	31	*Ae. aegypti*
17	−	2.19	AAEL008787_b	231	V-ATP synthase subunit alpha	5.26	68.5	42	*Ae. aegypti*
18	−	1.82	AAEL008787_c	132	V-ATP synthase subunit alpha	5.26	68.5	30	*Ae. aegypti*
27	−	1.51	AAEL005798_a	200	V-ATP synthase subunit beta	5.31	54.8	49	*Ae. aegypti*
28	−	1.69	AAEL005798_b	177	V-ATP synthase subunit beta	5.31	54.8	44	*Ae. aegypti*
29	−	1.72	AAEL005798_c	187	V-ATP synthase subunit beta	5.31	55.4	53	*Ae. aegypti*
30	−	2.06	AAEL005798_d	229	V-ATP synthase subunit beta	5.38	55.5	52	*Ae. aegypti*
31	−	2.39	AAEL005798_e	197	V-ATP synthase subunit beta	5.38	55.4	48	*Ae. aegypti*
32	−	1.89	AAEL005798_f	181	V-ATP synthase subunit beta	5.38	55.4	56	*Ae. aegypti*
52	−	2.14	AAEL012035_a	93	V-ATP synthase subunit E	5.91	25.7	35	*Ae. aegypti*
53	−	2.21	AAEL012035_b	88	V-ATP synthase subunit E	5.91	25.7	38	*Ae. aegypti*
*Other functions*									
8	+	10.74	AAEL001005_a	195	calreticulin	4.42	47.0	43	*Ae. aegypti*
8	−	2.56	AAEL001005_a	226	calreticulin	4.42	47.0	49	*Ae. aegypti*
9	+	3.14	AAEL001005_b	210	calreticulin	4.42	47.0	49	*Ae. aegypti*
10	+	2.75	AAEL001005_c	158	calreticulin	4.42	47.0	48	*Ae. aegypti*
12	+	3.01	AAEL001005_d	93	calreticulin	4.42	46.7	35	*Ae. aegypti*

Proteins are classified according to their putative function using the same 13 categories as for transcriptomic data. When different spots pointed to the same protein, they were differentiated using letters after the access number. For each identification, the predicted pI, the predicted mass in kiloDaltons (kDa), the percentage of sequence coverage and the species and database matched are indicated.

Two alkaline phosphatases (ALP) proteins, matching AAEL003313 and AAEL003298, were under-expressed in the LiTOX strain while transcriptomics identified two other ALP genes (AAEL011175 and AAEL015070) significantly over-transcribed in the LiTOX strain with transcription ratios of +4.63 and +3.95 fold respectively. All ALPs but AAEL003298 have predicted glycosylphosphatidylinositol (GPI)-anchor domains allowing them to tether to the epithelial membrane and be potential membrane-bound Cry toxin receptors (Additional file 6).

Four proteins matching ATP synthase subunits alpha, beta and epsilon, with two to seven different spots for the same protein, had levels from −1.51 to −2.39 lower in the LiTOX strain. This tendency was consistent with microarray data for AAEL008787-RA (−1.19 fold), AAEL005798 (−1.37 fold) and AAEL012035 (−1.19 fold) although P values were not significant.

A unique calreticulin protein was picked and identified from DIGE gels. Initially detected as only one big spot with +10.74 fold change, the second biological replicate allowed clearly identifying four different spots respectively −2.56, +2.75, +3.01 and +3.14 fold differentially expressed in the LiTOX strain. In microarray experiment, no significant differential transcription of this gene was found in the LiTOX strain.

Using BLASTP software, we managed to identify putative functions for the 15 transcripts of unknown function differentially transcribed in the LiTOX strain with protein identities ranging from 25 to 99% (Additional file 2). Among them, two were strongly over-transcribed in the resistant strain (AAEL013584 19-fold and AAEL010435 9.6-fold) and matched to a putative G_12_ protein in *Ae. aegypti* (77% protein identity, Additional file 2).

Among the five cytochrome P450 monooxygenase transcripts identified by microarray analyses, *CYP4D24* was over-transcribed while the others (*CYP6N9*, *CYP6Z7*, *CYP6Z8* and *CYP9M9*) were under-transcribed in the LiTOX strain. DIGE experiments identified one protein matching to a cytochrome P450 3-fold over-expressed in the LiTOX strain.

Transcriptomic data detected four metalloproteinases significantly under-transcribed from −3.16 to −5.29 fold (Additional file 2). The presence of conserved domains of soluble astacin-like metalloproteinases together with the absence of detected GPI-anchor domain (Additional file 6) suggests that these four metalloproteinases are probably secreted extracellular enzymes, explaining why they were not identified in the BBMV by the DIGE analysis.

### Global and specific proteolytic activities

To determine if the modifications in protease transcription levels observed in the resistant strain result in changes in gut proteolytic activities, we compared the protease activities of secreted proteins from larval midgut of each strain using azocasein as substrate. Total proteolytic activity was 8.5% higher in the resistant strain compared to the susceptible strain (Table 3). The use of specific protease inhibitors revealed that more than 90% of the total proteolytic activity is due to serine protease for both strains. Among them, chymotrypsins and trypsins represented respectively more than 40% and 20% of the total activity in both strains. The use of the metalloproteinase inhibitor EDTA showed that 12% of the total proteolytic activity was due to metalloproteinases in the susceptible strain whereas no inhibition was measured in the resistant strain (Wilcoxon test; P-value <0.05), suggesting a strong reduction of metalloproteinase activity in the LiTOX strain.

**Table 3:** Total enzymatic activity and effect of protease inhibitors on the azocaseinolytic activity of midgut extract

**Strain**	**Total enzymatic activity (OD at 440 nm)**	**Percentage of inhibition of total activity (%)**			
		**PMSF**	**TLCK**	**TPCK**	**EDTA**
Susceptible	0.328 ± 0.009	92.98 ± 0.57	46.82 ± 1.02	23.42 ± 2.24	12.18 ± 1.74
LiTOX	0.356 ± 0.010	92.31 ± 0.23	42.01 ± 2.29	20.87 ± 1.64	−1.74 ± 4.55
Wilcoxon test	*	NS	NS	NS	*

All values are given as mean +/− SEM.

## Discussion

### Resistance levels to *Bti* toxins in the LiTOX strain

After 30 generations of selection, resistance to Cry4Aa in the LiTOX strain has more than doubled as compared to twelve generations ago, while resistance ratios did not change for Cry4Ba and Cry11Aa [19,22]. Resistance to *Bti* is moderate (3.5 fold) but higher than at generation 18 (2-fold), indicating that resistance alleles are not all fixed yet. These results are consistent with previous attempts to select *Ae. aegyptiCulex pipiens* and *Cx. quinquefasciatus* with *Bti* which obtained moderate resistance (2 to 3 fold) after 20 to 30 generations [20,21,25,26]. The increased *Bti* resistance observed may be due to the increase in Cry4Aa resistance, and most changes observed in the present study may be related to Cry4Aa resistance. The discrepancy between *Bti* and Cry4Aa increased resistances is likely to be due to the presence of Cyt toxin in *Bti*, known to overcome Cry resistance in insects [15].

### Midgut transcriptome and proteome analyses

Our comparison of midgut transcripts and brush border proteins between the susceptible and LiTOX strains revealed an overlapping but distinct set of transcripts/proteins differentially expressed. Transcriptome profiling with a microarray representing more than 81% of known *Ae. aegypti* transcripts lead to the detection of 3512 transcripts of which 70 were differentially transcribed in the LiTOX strain. This relatively low number of transcripts detected (about 25%) is probably due to the low transcription level or absence of transcription of several genes in this particular organ —the larval midgut. Little overlap was observed between the previous transcriptomic analysis, performed on whole larvae 12 generations ago using a DGETP approach [23], and the present study, focusing on midgut gene expression using microarrays. This is possibly due to the technical differences between the two studies and to the fact that no resistance gene is fixed yet in the LiTOX strain, indicated by the still increasing resistance to *Bti* and to Cry toxins [19]. Moreover, as genes conferring resistance to *Bti* toxins are likely to be expressed in larval midgut, focusing on midguts rather than on whole larvae allows to considerably reduce the candidate gene dataset, and to consider only genes likely to be directly involved in resistance, rather than those only indirectly affected by selection side-effects (genetic drift) or compensatory mechanisms (resistance costs) [24]. The 2D-DIGE analysis resolved about 400 distinct proteins in larval BBMV fractions, 56 spots were picked of which 50 differed by more than 2-fold between the two strains. The difference between the number of spots picked (56) and the number of unique identified proteins (35) is due to different spots for the same protein, as for example up to six spots observed for one V-ATPase. The multiple spots for the same protein are most likely due to post-translational modifications (glycosylation, phosphorylation) that cause shift in protein mobility. Eight spots common to the replicated DIGE experiments were picked and identified twice. Among them all but one, calreticulin (AAEL01005), showed similar levels of differential expression supporting the consistency of biological replicates (Additional file 4). Both transcriptomic and proteomic data identified more under than over-expressed genes/proteins in the LiTOX strain, which is congruent with a previous transcriptome analysis performed on whole larvae 12 generations ago [23]. Such asymmetry is not surprising considering that mechanisms of resistance to *Bt* can involve a decreased activation of protoxins or a decreased toxin-binding to the epithelium membrane [27].

Little overlap was found between data obtained by transcriptomic and proteomic approaches. This could be explained by both biological processes and technical limitations inherent to each method. Regarding DIGE, BBMV were used, which are enriched for proteins attached to apical brush border midgut membrane via scaffolding and proteins attached to the inner membrane leaflet via acylation. Therefore, except few soluble proteins trapped in re-folded membranes, soluble intracellular proteins and proteins excreted inside the gut lumen are typically absent in BBMV preparations [28,29]. In contrast, mRNAs extracted from whole larval midguts should be representative of all transcripts present in midgut cells. Another factor limiting overlapping data may be the consequence of the relatively stringent filtering of the microarrays dataset (3-fold threshold). Several studies also showed that mRNA transcription profiles fit poorly with protein levels because of numerous post-transcriptional regulatory activities and post-translational events [30-32]. Such events generate a high diversity of proteins while gene expression remains unchanged, and this source of variation is so far under-explored in studies on fast adaptive changes like the evolution of insecticide resistance. It is likely that the two complementary approaches used in the present work detected distinct mechanisms of resistance acting at different steps in the mode of action of *Bti* (i.e. crystal solubilization, toxin activation and binding to receptors).

### Altered expression and activities of proteases from the LiTOX strain

Four soluble astacin-like metalloproteinases were found significantly under-transcribed in the LiTOX strain. This observation was correlated with a strong decrease of metalloproteinases activity among the enzymes secreted in the midgut lumen of larvae from the resistant strain. To our knowledge, this is the first time astacin-like metalloproteinases are associated to *Bt* resistance. The observed decrease in metalloproteinases in the resistant strain might reflect an alteration in *Bti* Cry toxins activation in the gut lumen of LiTOX larvae. Further experiments based on measuring proteolytic activities and performing bioassays with activated toxins will clarify the potential role that alteration of protoxins processing, notably for Cry4Aa, could play in the resistance phenotype.

### Altered expression of known *Bti-*binding proteins in the LiTOX strain

To validate the expression alteration of putative *Bti*-receptors observed in microarrays and DIGE approaches, RT-qPCR analyses were performed on five N-aminopeptidases (APN1 to 5), two cadherins (Cad1 and Cad2) and three alkaline phosphatases (ALP1 to 3) previously described as binding proteins for Cry4Ba [29] or Cry11Aa [33-36] (Table 4). 

**Table 4:** **Altered expression of known*Bti*Cry-binding proteins detected by transcriptomic and proteomic approaches**

**Gene**	**Accession number**	**Microarrays**	**RT-qPCR**	**2D-DIGE**	**Binding protein**	**Ref**
Alkaline phosphatase (ALP1)	AAEL000931	ND	+11.40	NI	Cry11Aa	[33]
Alkaline phosphatase (ALP2)	AAEL003298	−1.57	−1.56	−1.92; −1.16	Cry4Ba	[29]
Alkaline phosphatase (ALP3)	AAEL003313	+1.15	+1.52	−1.58	Cry4Ba	[29]
Alkaline phosphatase (ALP4)	AAEL009077	ND	+1.15	NI	Cry11Aa	[36]
Alkaline phosphatase (ALP5)	AAEL015070	+3.95	+7.69	NI	Cry4Ba	[29]
Cadherin (Cad1)	AAEL007478	ND	−1.11	UD	Cry11Aa	[34]
Cadherin (Cad2)	AAEL007488	−1.47	−1.46	UD	Cry11Aa	[34]
N-Aminopeptidase (APN1)	AAEL012774	+1.44	+1.26	+1.80; +2.34	Cry11Aa	[35]
N-Aminopeptidase (APN2)	AAEL012776	−1.34	−1.62	+1.81; −2.74; −3.81	Cry4Ba	[29]
N-Aminopeptidase (APN3)	AAEL012778	−1.04	+1.20	NI	Cry11Aa	[35]

Given values indicate the level of expression in the LiTOX strain compared to the susceptible strain detected in microarrays, RT-qPCR and DIGE experiments. ND, Non detected in at least 5 of the 6 microarray hydribizations; NI, Non identified as differentially expressed between the two strains and therefore non-picked for MS/MS identification; UD, Undetectable in 2D-DIGE due to their high molecular weight and their low amount in BBMV.

The cadherin described as a Cry11Aa-receptor in *Ae. aegypti* (AAEL007488) [34] was found 1.47 fold under-transcribed in both microarrays and RT-qPCR experiments. However, no cadherin was detected by DIGE approach. The inability to detect cadherin in the DIGE analysis is not surprising as they are large proteins (>170 kDa) present in low amounts in insect brush border membranes [37]. Blotting of BBMV using two anti-cadherin antibodies showed that most of the cadherins were degraded, even in a freshly prepared UGAL *Aedes* BBMV preparation, confirming that cadherins in BBMV are very unstable (Additional file 7). Western blots showed that cadherin(s), notably a ~32 kDa fragment, is strongly over-represented in the LiTOX strain compared to the susceptible strain (Additional file 7). Further analyses of the toxins-binding properties of the detected cadherin(s) are needed to better understand the role they could play in the resistance phenotype.

Alkaline phosphatases (ALPs), typically anchored by GPI-moieties, are known to be Cry toxin receptors in Lepidoptera [38,39] and mosquitoes [36,40]. Recently, a decrease in ALP amounts and activities were linked to Cry-resistance of larvae from three lepidopteran genera [41]. The ALPs detected as over-transcribed in the LiTOX strain by the two transcriptomic approaches were either not identified as differentially expressed (AAEL000931, AAEL009077 and AAEL015070) or identified as under-expressed (AAEL003313) by DIGE approach. These results suggest that the lower ALP protein abundance in the epithelium membrane might rather be due to post-translational events than under-expression. Indeed, our DIGE analyses identified three ALPs showing a decreased expression in the LiTOX strain (AAEL003313 and two spots of AAEL003298). Moreover, the ALP AAEL003298 was also detected as under-transcribed in microarrays, RT-qPCR and in a previous transcriptomic study [23]. These two ALP have already been described as Cry4Ba-binding proteins [29]. The reduction of potential Cry-receptor ALPs proteins on the brush border of LiTOX larvae is consistent with the resistant phenotype.

N-Aminopeptidases (APNs) are a third major class of Cry toxin receptors in Lepidoptera [42] and mosquitoes [43,44] and their alteration correlates with Cry1A-resistance in *Helicoverpa armigera*[45] and *Trichoplusia ni*[46]. DIGE experiments revealed two spots of APN AAEL012776 being under-expressed, congruent with transcriptomic data, while another spot was over-expressed in the LiTOX strain. Two spots matching APN AAEL012774 were over-expressed in the LiTOX strain, as also found by transcriptomic approaches. These two ALP proteins have been previously described as potential receptors for Cry11Aa in *Ae. aegypti*[35] but it is still unclear how their altered expression could lead to a higher *Bti*-resistance.

In general, *Bt* resistance involves changes in the Cry receptors structure rather than in their expression [27,47], although some cases of differential expression of cadherin and aminopeptidase have been reported in resistant strains [48-50]. These changes in expression can be the result of diverse genetic mechanisms including mutations in regulatory regions or even genome rearrangements that can drive rapid adaptation to new environmental pressures such as an insecticide treatment. Moreover, in the case of *Bti*, the presence of Cyt toxins, known to act as Cry receptors [51], might contribute to overcome receptor alterations in the LiTOX strain. Further analysis of the binding capacities of Cry toxins to the putative receptors found differentially expressed here will contribute to evaluate their relative roles in *Bti* resistance. Only few studies have focused on Cry4Aa toxin binding to our knowledge [52,53] and nothing is known about its potential membrane receptors. Such experiments will determine if its receptors are highly specific, explaining the high differences in the resistance ratio between Cry4Aa and the other Cry toxins in the LiTOX strain, or if Cry4Aa shares all or a part of its receptors with Cry4Ba and Cry11Aa that could lead to cross-resistance.

### Other mechanisms potentially involved in the resistance

All the spots of the four ATP synthases detected by our proteomic approach showed an under-expression pattern in the LiTOX strain. Vacuolar H^+^-ATPases (V-ATPase) subunits B to E are known to bind Cry4Ba [29] and subunits A and B have been described to bind for Cry1Ac in *Heliothis virescens*[54] and *Helicoverpa armigera*[55]. Moreover, V-ATPases are localized in the posterior midgut of mosquito larvae [56], where Cry4Aa, Cry4Ba and Cry11Aa toxins exhibit the highest affinity to the epithelium membrane [52,53,57,58]. Nevertheless, their role as *Bti* toxins receptors has not been demonstrated yet. V-ATPases are strongly implicated in the alkalinization of the midgut pH by establishing a proton motive force by transporting proton across membranes leading to a pH gradient and transmembrane voltage [59-61]. Onken *et al.* (2008) inhibited all the proteins implicated in the alkalinization process in the midgut of *Ae. aegypti* larvae and they showed that only the inhibition of V-ATPases induced a strong acidification of the midgut pH [62]. As pH affects numerous aspects of toxin action like *Bt* crystal solubility [7], toxin conformation [63,64], gut enzymes activity [65] and pore formation [66,67], an alteration of gut pH could have a general effect on reducing *Bti* toxicity. Comparing internal larval midgut pH between resistant and susceptible strains will allow to confirm/infirm if the observed ATPases decreased expression induce an acidification of the gut lumen.

Multiple detoxification enzymes were found under-transcribed in the resistant strain. Such enzymes are often involved in the degradation of small chemicals such as insecticides and plant allele-chemicals [68,69], but they are unlikely to process large proteins such as *Bti* toxins. Synthesis of detoxification enzymes represents an important energetic cost for the insect [70]. Moreover, several detoxification genes found under-transcribed in our dataset, were found over-transcribed in *Ae. aegypti* larvae submitted to a chemical challenge [71]. Although the over-expression of particular detoxification genes in the resistant strain can be linked to larval response to tannins contained in the toxic leaf litter [72], the frequent under-expression of these enzymes in the resistant strain may reflect compensatory mechanisms.

## Conclusion

*Bti* has evolved to infect Diptera such as mosquitoes and blackflies through a sequential mechanism. The multi-step mode of action of *Bti* and its toxins from ingestion to spore germination and proliferation offers many resistance ways for mosquito larvae. By combining transcriptomic and proteomic approaches, we detected expression alteration at nearly each step of the ingestion-to-infection process. Our study paves the way to further functional studies to characterize resistance mechanisms to this bioinsecticide. This information will be of extreme value as this environmentally safe bioinsecticide is increasingly used for vector control worldwide with virtually no knowledge and no suspicion so far about how mosquitoes can develop resistance in the field.

## Methods

### Mosquito strains

The *Ae. aegypti* laboratory strain *Bora-Bora*, susceptible to all insecticides, was used for selection with field-collected leaf litter containing *Bti* spores and toxins [16]. This material, highly toxic after ingestion by mosquito larvae, was used for laboratory selection during 30 generations to obtain the LiTOX strain. Selection consisted in exposing 6000 third instar larvae to toxic leaf litter to obtain about 70% of larval mortality after 48 h exposure [19]. Both susceptible and resistant strains were reared in standard insectary conditions (27°C, 14/10 h light/dark period, 80% relative humidity). Larvae were reared in tap water and fed with standard amount of larval food [19,73].

### Production of individual *Bti* Cry toxins

To produce *Bti* Cry toxins separately, we used a crystal negative strain of *Bacillus thuringiensis* var. *israelensis* (4Q2-81) transformed with the plasmids pHT606, pHT618 or pWF53 producing respectively Cry4A, Cry4B and Cry11 toxins obtained from the Pasteur Institute (Paris, France) or from Prof. B. Federici (University of Riverside, USA). Transformed *Bti* bacteria were grown on Nutrient Agar solid medium (Sigma Aldrich) supplemented with erythromycin antibiotic (25 μg/mL). Spores and crystals were recovered using cell scrapers (BD Falcon) after 7 days at 30°C and purified as previously described [19]. This protocol ensures producing large amount of high quality toxin. Toxins were corun on SDS-PAGE with BSA at five concentrations (from 20, 40, 60, 80, 100 μg/mL). Intensity of each band was estimated and toxin concentration was calculated using BSA as standard using Imagej software v.1.41o [74].

### Bioassays

Comparative bioassays between the LiTOX and the susceptible Bora-Bora strains were conducted after 30 generations of laboratory selection. Larvae from each strain were exposed to 6 concentrations of Cry4Aa, Cry4Ba, Cry11Aa and commercial *Bti* (Vectobac WG, 3500 ITU/mg) for 24 h to obtain 5% to 95% mortality. Bioassays were performed in triplicate on 20 third-instar larvae in 50 mL of insecticide solution or tap water (control) according to the standard bioassay procedure described by the World Health Organisation [75]. LC_50_ (lethal concentration for 50% individuals) were calculated for each strain and each toxin using a probit statistical model with the module ‘dose’ of XLSTAT v.2009.4.06 (Addinsoft). For each toxin, resistance ratios (RR_50_) were calculated by dividing LC_50_ of the LiTOX strain by LC_50_ of the susceptible strain.

### Larval midgut RNA extraction

For each strain, three biological replicates of 150 dissected midguts from early fourth instar larvae were prepared and conserved overnight at 4°C in RNA*later®* (Ambion). After a brief centrifugation, supernatant was discarded and total RNA was extracted using RNAqueous®-4PCR kit (Ambion) following manufacturer’s instructions. Quantity and quality of RNA were assessed by spectrophotometry (Nanodrop ND-1000 spectrophotometer). To digest remaining genomic DNA, RNA samples were treated with DNAseI (Ambion) following manufacturer’s instructions. RNA were then concentrated using ammonium acetate and linear acrylamide to obtain at least 70 ng/μL of total RNA for each sample. Because the LiTOX strain was selected from the Bora-Bora strain, they share the same genetic background, and both were bred together in the same insectarium standard conditions, so that any constitutive change in gene expression between these strains is likely to result from *Bti* selection.

### Larval midguts transcriptome profiling by DNA microarray

Low Input Quick Amp Labeling Kit, two-color (Agilent), containing Cy5 and Cy3 fluorescent dyes, was used to amplify and label messenger RNA. Labeled RNAs were then purified using Absolutely RNA® Nanoprep Kit (Stratagene) following manufacturer’s instructions with two elution steps in a final volume of 25 μL. Quantity and quality of RNA and labeling efficiency were assessed using Nanodrop spectrophotometer and Bioanalyzer® (Agilent).

Microarray hybridizations were performed with the 15 K Agilent ‘*Aedes detox chip plus*’ DNA microarray (ArrayExpress accession number A-MEXP-1966), containing eight replicated arrays of 60-mers oligo-probes representing 14204 different *Ae. aegypti* transcripts and several control probes. For each biological replicate, two hybridizations were performed in which the Cy3 and Cy5 labels were swapped between samples for a total of six hybridizations. For each hybridization, 300 ng of labeled mRNA were used. After 17 h hybridization, non-specific probes were washed off according to manufacturer’s instructions. Slides were scanned with an Agilent G2205B microarray scanner. Spot finding and signal quantification were performed using the Agilent Feature Extraction software (Agilent Technologies).

Data were analyzed using GeneSpring GX v9.0 software (Agilent). Only transcripts present in at least 5 hybridizations out of 6 were kept for further analyses. Transcripts exhibiting more than 3-fold transcription and a Benjamini-Hochberg [76] corrected P-value <0.01 were considered significantly differentially transcribed between the LiTOX and the susceptible strain. Midgut transcripts detected by microarrays were then classified into thirteen different categories based on their putative biological functions: receptors, transport, DNA interaction, cytoskeleton, ribosomal proteins, proteases, detoxification enzymes, kinases-phosphatases, transaminases, dehydrogenases, other enzymes, other function and unknown function. For genes of ‘unknown function’, the putative function was further investigated using BLASTP software, but they were not considered for functional analysis.

### Real-time quantitative PCR (RT-qPCR) validation of microarray data

Transcription levels of 15 genes detected differentially transcribed with the microarray approach were validated by RT-qPCR using the same RNA extracts used in microarrays. In addition, transcription levels of two more genes (ALP1 and Cad1) encoding known *Bti* Cry toxins binding proteins were also compared between both strains by RT-qPCR. Three technical replicates were performed for each of the three biological replicates. Specific primers were designed for each gene using Beacon Designer v.5.10 software (Premier Biosoft International) (Additional file 8). Their specificity to the target gene was verified by BLAST analysis against *Ae. aegypti* genome. First-strand cDNA synthesis was obtained from 4 μg RNA by incubating them at 50°C for 1 h with SuperScript III (Invitrogen) reverse transcriptase, oligo-dT_20_ primers (2.5 μM), dNTPs (0.5 mM each), DTT (5 mM) and RNase Out (40 U, Invitrogen). Real-time quantitative PCR reaction was performed in 25 μL total reaction volume with specific primers (0.3 μM each), 12.5 μL iQ SYBR Green supermix (Bio-Rad) and 5 μL diluted cDNA on an iQ5 system (Bio-Rad). After an initial denaturing step at 95°C for 3 min, 40 cycles were performed each consisting in a denaturing step 15 s at 95°C and an annealing step 30 s at the optimal temperature of each primers couple (Additional file 8) [22,71]. Specificity of DNA amplification was assessed by performing a melt curve analysis and verifying PCR product Tm. To check for any contamination, “no template controls” (NTC) were added in each PCR plate.

For each gene analyzed, a serial dilution of pooled cDNA from both strains was used to estimate PCR efficiency. Genes encoding ribosomal proteins RPL8 and RPS7 (housekeeping genes) were used for gene expression normalization taking into account PCR efficiency using ΔΔC_T_ method, calculated using the iQ5 software (Bio-Rad) [77,78]. Mean transcription ratios are expressed for the resistant strain relative to the susceptible strain.

### GPI-anchor domain detection

To see whether some midgut enzymes detected with DNA microarrays were membrane-bound, we looked for glycosylphosphatidylinositol (GPI)-anchor domains using four complementary GPI domains predictors: big-PI Predictor v.3.0 (http://mendel.imp.ac.at/gpi/gpi_server.html) [79], PredGPI (http://gpcr.biocomp.unibo.it/predgpi/pred.htm) [80], FragAnchor (http://navet.ics.hawaii.edu/~fraganchor/NNHMM/NNHMM.html) [81] and GPI-SOM (http://gpi.unibe.ch/) [82].

### Brush border membrane vesicles (BBMV) preparation

For each strain, two independent biological replicates were prepared. The day before midgut dissection, water was changed and food discarded. Early fourth instar larvae were chilled on ice for at least 20 min. Larvae were then dried on a clean paper. Midguts were dissected and mixed together in MET buffer (300 mM Mannitol, 5 mM EGTA, 17 mM TrisHCl, pH 7.5) with Complete Protease Inhibitor (Roche) to be conserved at −80°C until use. About 1500 larvae were dissected for each larval strain and biological replicate. 500 μg of midguts were centrifuged 5 min at 12,000 g to discard the old buffer, resuspended in ice-cold fresh MET buffer containing 1 mM PMSF and homogenized with 30 strokes of a glass-teflon homogenizer. BBMV were prepared following magnesium precipitation method as previously described [83]. BBMV protein concentration was determined by a Bradford assay using BSA as standard [84]. About 600 dissected guts yielded 500 μg of BBMV based on protein amount. Quality of BBMV was assessed by measuring the enrichment of two brush border enzymes: alkaline phosphatases (ALP) and aminopeptidases (APN). ALP and APN activities were measured using 4-nitrophenyl phosphate disodium and L-leucine-p-nitroanilide as substrates, respectively [85,86]. APN and ALP enrichments are obtained by dividing the activity in the final BBMV preparation by the activity in the initial midgut homogenate (Table 5). 

**Table 5:** APN and ALP enrichments in final BBMV preparation relative to the initial midgut homogenate

**Strain**	**Biological Replicate**	**APN enrichment**	**ALP enrichment**
Susceptible	First	4.4 fold	6.1 fold
	Second	5.3 fold	1.6 fold
LiTOX	First	5.0 fold	7.1 fold
	Second	4.8 fold	3.2 fold

Enrichments are given for each of the two biological replicates for each strain.

### 2D-DIGE

150 μg of BBMV proteins from each strain were used for each 2D-DIGE experiment and were purified using 2D-clean up kit (Amersham Bioscience) as described by the manufacturer. 100 μg of proteins were labeled with either Cy3 or Cy5 and the remaining 50 μg of proteins from the each strain were pooled and labeled with Cy2 as an internal standard. A dye swap was performed to be sure that the observed differences between the two strains were not due to different efficiencies of the dyes to label different proteins. The CyDye minimal labeling of the purified proteins was performed following manufacturer’s instructions (GE Healthcare). Labeled proteins were then mixed together and diluted to a final volume of 340 μL with rehydration buffer (2 M Thiourea, 7 M Urea, 3% CHAPS, 1% SB3-10, 13 mM DTT, 1% Immobilized pH Gradient (IPG) buffer pH 4–7, 0.002% Bromophenol blue (w/v)) and loaded on an IPG strip (pH 4–7 nonlinear, 18 cm) overlaid with 2 mL of plus-one IPG strip cover fluid (GE Healthcare). After 17 h of passive rehydration, the first dimension was run on a Multiphor-II flatbed system (GE Healthcare) at 20°C with the following program: 15 min at 300 V, 15 min at 500 V, and 9 h at 3500 V. This step allows proteins to migrate on the strip till a region in which pH is equal to their pI (isoelectric point).

After the Isoelectric Focusing, strips were reduced in equilibration buffer (6 M Urea, 75 mM Tris pH 8.8, 2%SDS, 29.3% Glycerol (v/v), 0.002% Bromophenol blue (w/v)) containing 1% of DTT (w/v) for 15 min and then alkylated in equilibration buffer with 2.5% of Iodoacetamide for 15 min. The IPG strip was then transferred on a pre-casted 12.5% SDS-PAGE gel (GE Healthcare) and second dimensional electrophoresis, separating proteins in function of their molecular size, was run at 22°C for 1 h at 2.5 W/gel followed by 5 h at 17 W/gel on an Ettan DALT*six* vertical electrophoresis system (GE Healthcare). DIGE Gels were scanned using a Typhoon 9400 imager (GE Healthcare). As CyDye labeling induces a size modification of 1–2% of the amount of all the proteins that could bias the protein identification, non-labeled proteins were also prepared in parallel following the same protocol (except CyDye labeling) for mass spectra analyses and regular gels were co-run with DIGE gels to avoid modification in spot patterns due to different migrations. Regular gels were stained with Deep Purple stain (GE Healthcare), scanned using 532/610 nm excitation/emission wavelengths and used for spot picking.

### Protein identification

2D-DIGE gels were analyzed using Decyder v7.0 software (GE Healthcare). The Decyder detection algorithm 5.0 was used to generate a list of spots with their coordinates and level of expression in the resistant strain relative to the susceptible strain. Only spots showing at least 1.5 fold differences between the two strains were considered for further analyses. 29 spots were picked from the first biological replicate and 35 from the second one, with 8 spots shared between them. Excised spots were digested with trypsin before subjecting peptides fragments to MALDI-ToF/ToF (time-of-flight) [29]. To increase the likelihood of protein identification, each protein was identified by searching MS/MS data against an *Ae. aegypti* local database or other dipteran database when no significant match was obtained. To ensure accurate protein identification, we compared observed and expected pI values, molecular size, percentage of amino acid coverage and Mascot scores for Mascot search engine (http://www.matrixscience.com/search_intro.html) or z-scores for ProFound (http://prowl.rockefeller.edu).

### Cadherin detection by immunoblotting

20 μg of proteins from BBMV prepared from the susceptible Bora-Bora strain, the LiTOX strain and the UGAL strain were separated by SDS-PAGE on 4–20% gradient TGX gels (Biorad). BBMV from the UGAL strain were prepared a few days before the experiments to compare the cadherin conservation in those fresh BBMV to the previously prepared BBMV from the two other strains. Proteins were either stained with coomassie blue to control that equal amount of proteins were stained from all the strains, or electroblotted to polyvinylidene fluoride (PVDF) filters for immunoblotting. Filters were blocked with 3% bovine serum albumin (BSA) in PBST (PBS + 0.1% Tween20) for 1 h at room temperature and then probed with α-AgCad1 antibodies (1:5000 dilution) [87], α-AgCad2 antibodies (1:500 dilution) or with pre-immune serum from the AgCad2 rabbit in PBST-0.1% BSA for 2 h. α-AgCad2 antibodies were prepared against an *E. coli* expressed cadherin peptide AgCad2 (Hua *et al.*, unpublished work). Filters were then washed and detected by an anti-rabbit IgG-peroxidase conjugate (1:25000 dilution) in PBST-0.1% BSA for 1 h at room temperature. Filters were developed with an ECL kit (GE Healthcare) and chemiluminescence was detected with a ChemiImager (Alpha Innotech). All the Western blots were performed in duplicate.

### Larval midgut proteolytic activities

For each strain, three biological replicates of soluble protein extracted from midgut juice were prepared. 20 midguts of early fourth instars were extracted and placed into 50 μL of distilled water and homogenized using a vortex for 30 s. Sample were centrifuged at 12,000 g for 10 min at 4°C. All the supernatants from larvae of the same biological replicate were mixed together, protein concentration was quantified by a Bradford assay using BSA as standard [84] and aliquots were conserved at −20°C until use. Total protease activity was measured using azocasein as substrate (Sigma Aldrich) as described in [88]. All activities were normalized according to the amount of total protein from each replicate. For each biological replicate, six technical replicates were performed and absorbance was measured at 440 nm. Percentages of protease activity due to serine proteases, chymotrypsins, trypsins and metallo-enzymes were measured using respectively PMSF (30 mM), TPCK (1.5 mM), TLCK (1.5 mM) and EDTA (1 mM) (Sigma Aldrich) [88]. Statistical differences between the two strains were measured by a Wilcoxon test performed with R 2.8.1 software [89].

## Abbreviations

ALP: Alkaline phosphatase; APN: N-aminopeptidase; BBMV: Brush border membrane vesicles; DIGE: Differential in gel electrophoresis; EDTA: Ethylenediaminetetraacetic acid; GPI: Glycosylphosphatidylinositol; MS/MS: Tandem mass spectrometry; PMSF: Phenylmethylsulfonyl fluoride; PVDF: Polyvinylidene fluoride; TLCK: N_α_-tosyl-L-lysine chloromethyl ketone hydrochloride; TPCK: N-*p*-Tosyl-L-phenylalanine chloromethyl ketone.

## Competing interests

The authors declare that no competing interests exist.

## Author’s contributions

J. P.D., M.J.A. and L.D. designed research; G.T. and K.B. performed DiGE experiments; K.B. did Western blot experiments; G.T., K.B. and M.J.A. analyzed proteomic data; G.T. and M.A.R. prepared mRNA samples for microarrays and did gene expression analyses; C.M.J. performed microarray experiment; G.T. performed RT-qPCR experiments; G.T. and J.P.D analyzed transcriptomic data; G.T. and R.S. did enzymatic experiments; G.T. and M.P. performed the selection and rearing of the LiTOX strain; G.T. wrote the paper; K.B., R.S., J.P.D., M.J.A. and L.D. reviewed and helped improving the manuscript. All authors read and approved the manuscript.

## Author’s information

G.T. performed this study during his Ph.D. at the Laboratoire d’Ecologie Alpine (LECA), University of Grenoble. His research interests goes from the understanding of the fate of pesticides in the field to the adaptive strategies of target insects. K.B. worked on this study during his Ph.D. tenure in the Dept. of Entomology, University of Georgia. Currently, he is a Postdoctoral Research Associate in the Dept. of Microbiology, UGA. His research interest is Oxidative protein damage and protein repair in *Helicobacter pylori*. C.M.J. is a post-doctoral researcher in the Vector Group of the LSTM where his current interests lie in vector biology and genetics with a strong emphasis on insecticide resistance. R.S. participated to this study during his Master internship at the LECA. He is interested in molecular ecology and agricultural science. M.P. participated to this study during her Ph.D. She is now a post-doc in Zurich, and is interested in population genetics, molecular adaptation and evolution. J.P.D. is a senior CNRS researcher having a strong experience in vector control and adaptive mechanisms developed by insects to insecticides, pollutants and toxins. M.J.A., a professor at the University of Georgia and CSO of InsectiGen, investigates *Bt* toxins and their action in pest insects. L.D. is a Professor at University of Grenoble. Her main research interests are in adaptive patterns in natural populations and underlying evolutionary processes.

## Supplementary Material

Additional file 1 ** All the 3512 transcripts detected by microarrays experiments in at least 5 hybridizations out of 6.** For each transcript, accession number, corrected p-value, expression level changes, Vectorbase annotation and functional category are indicated. Click here for file

Additional file 2 ** 70 transcripts significantly (corrected P-val<0.01) more than 3-fold differentially transcribed in the LiTOX strain.** Transcripts are classified according to their putative function using the 13 functional categories. For each transcript, accession number, corrected P-value, expression level changes, Vectorbase annotation and supercontig are indicated. For transcripts of ‘unknown functions’, their putative function with corresponding score, ID, accession number and species of the best hit found using BLASTP software are indicated. Click here for file

Additional file 3 ** Validation of microarray data by RT-qPCR on fifteen selected genes. **Both experiments were performed on the same mRNA extracted from dissected larval midguts. ALP2, Alkaline phosphatase AAEL003298; ALP3, AAEL003313; ALP5, AAEL015070; ALP6, AAEL011175; APN1, N-Aminopeptidase AAEL012774; APN2, AAEL012776; APN3, AAEL012778; Cad2, Cadherin AAEL007488; HP1, Conserved hypothetical protein AAEL010435; HP2, AAEL013584; SE1, Serine-type endopeptidase AAEL007938; SE2, Serine-type endopeptidase AAEL011917; Cytochrome P450: *CYP6Z7*, AAEL009130; *CYP6Z8*, AAEL009131 and *CYP4D24*, AAEL007815. Click here for file

Additional file 4 ** 2D-DIGE gels from the two biological replicates and dye-swapping. **BBMV prepared from first (A and B) and second (C and D) biological replicate are separated in function of their size (kDa) and their isoelectric point (pI). BBMV from *Bti* resistant strain are labeled with Cy3 and susceptible strain with Cy5 (A and C) or resistant strain with Cy5 and susceptible with Cy3 (B and D). Click here for file

Additional file 5 **Protein identification of the 56 spots picked on deep purple stained 2D-gel.** When different spots pointed to the same protein, they were differentiated using arbitrary letters after the access number. For each identification, the predicted pI, the predicted mass in kilodaltons, the percentage of sequence coverage, their functional category, and the species and database matched are indicated. Click here for file

Additional file 6 ** Glycosylphosphatidylinositol (GPI)-anchor domains detection by four predictive computational programs.** For each gene and protein, their accession number, the transcript and protein sizes are indicated. Results from the big-GPI and GPI-SOM softwares are indicated as ‘YES’ when they found a potential GPI-domain and ‘NO’ when no GPI-domain was determined. For PredGPI, presence is indicated by ‘Highly probable’, ‘Weakly probable’ or ‘Probable’ and absence by ‘NO’. For FragAnchor, presence of GPI domain is indicated by ‘Highly probable’ or ‘Probable’, absence by ‘NO’ and when prediction is uncertain by ‘Potential false positive’. Click here for file

Additional file 7 **Cadherin detection by immunoblotting. **BBMV proteins from the susceptible Bora-Bora strain (lane 1), LiTOX strain (lane 2) and the UGAL *Aedes* strain (lane 3) were separated in SDS-PAGE and stained with coomassie blue (panel A) or probed with α-AgCad1 antibodies (panel B), α-AgCad2 antibodies (panel C) or with pre-immune serum from α-AgCad2 rabbit (panel D). Click here for file

Additional file 8 **Primer pairs used for RT-qPCR analyses.** For each primer pair, sequence, corresponding gene name and accession number, product length, Tm and optimal annealing temperature used in PCR program are indicated. PCR efficiency and different parameters of the calibration curves (R^2^, slope and y-intercept) are also indicated. Specificity of each primer pair was first assessed by BLAST analysis against *Ae. aegypti* genome and then verified by performing a melt curve analysis. A high specificity is indicated as “YES” when the primer pair matched to a unique position in the *Ae. aegypti* genome and when PCR product Tm was correct. Click here for file
