# Supplementary figures and images for: Larval midgut modifications associated with Bti resistance in the yellow fever mosquito using proteomic and transcriptomic approaches
Source: BMC Genomics. 2012 Jun 15;13:248. doi: 10.1186/1471-2164-13-248 (PMC3460780; doi:10.1186/1471-2164-13-248)

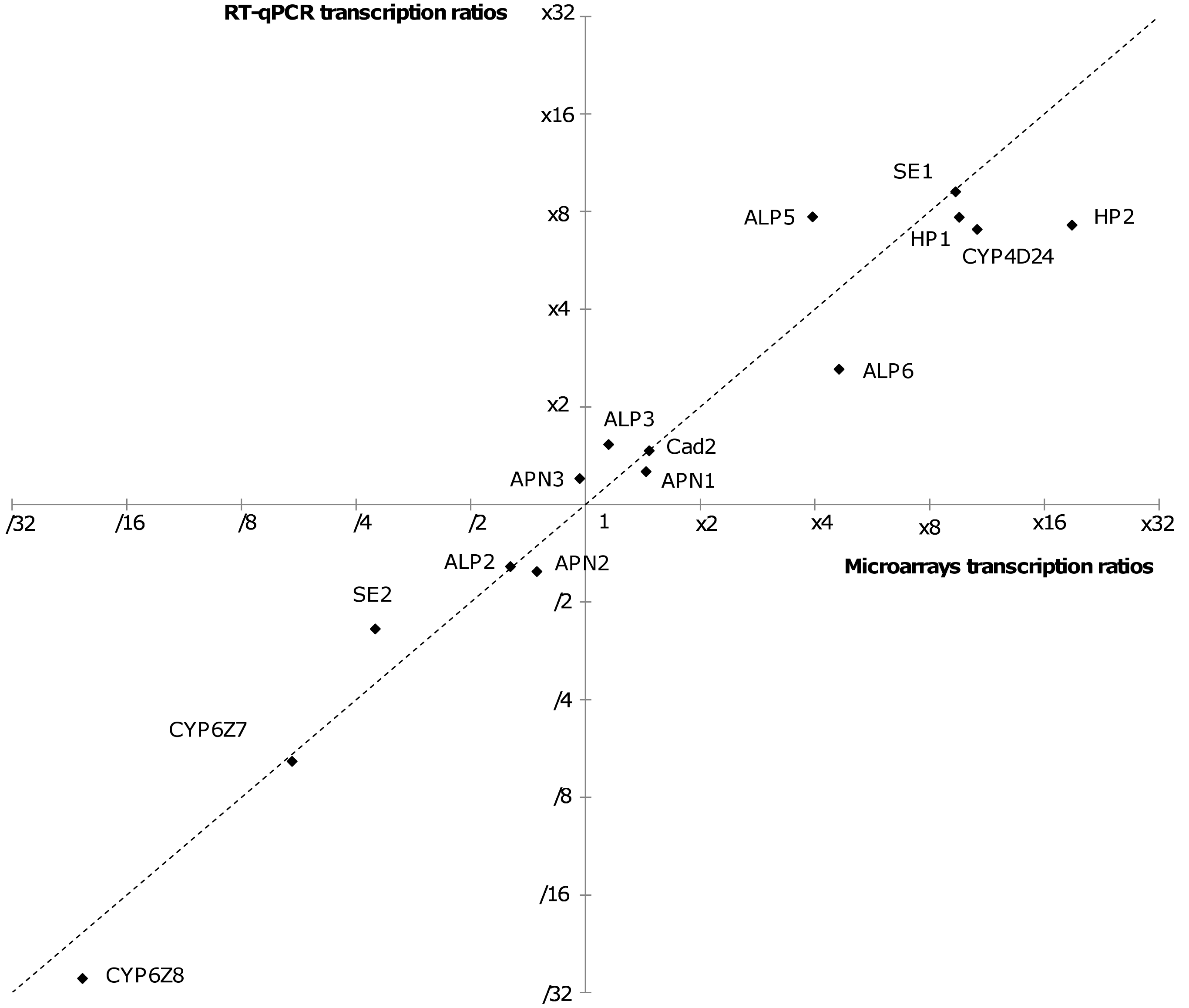

Supplement: Additional file 3 — Validation of microarray data by RT-qPCR on fifteen selected genes. Both experiments were performed on the same mRNA extracted from dissected larval midguts. ALP2, Alkaline phosphatase AAEL003298; ALP3, AAEL003313; ALP5, AAEL015070; ALP6, AAEL011175; APN1, N-Aminopeptidase AAEL012774; APN2, AAEL012776; APN3, AAEL012778; Cad2, Cadherin AAEL007488; HP1, Conserved hypothetical protein AAEL010435; HP2, AAEL013584; SE1, Serine-type endopeptidase AAEL007938; SE2, Serine-type endopeptidase AAEL011917; Cytochrome P450: CYP6Z7, AAEL009130; CYP6Z8, AAEL009131 and CYP4D24, AAEL007815. [file 1471-2164-13-248-S3.tiff]

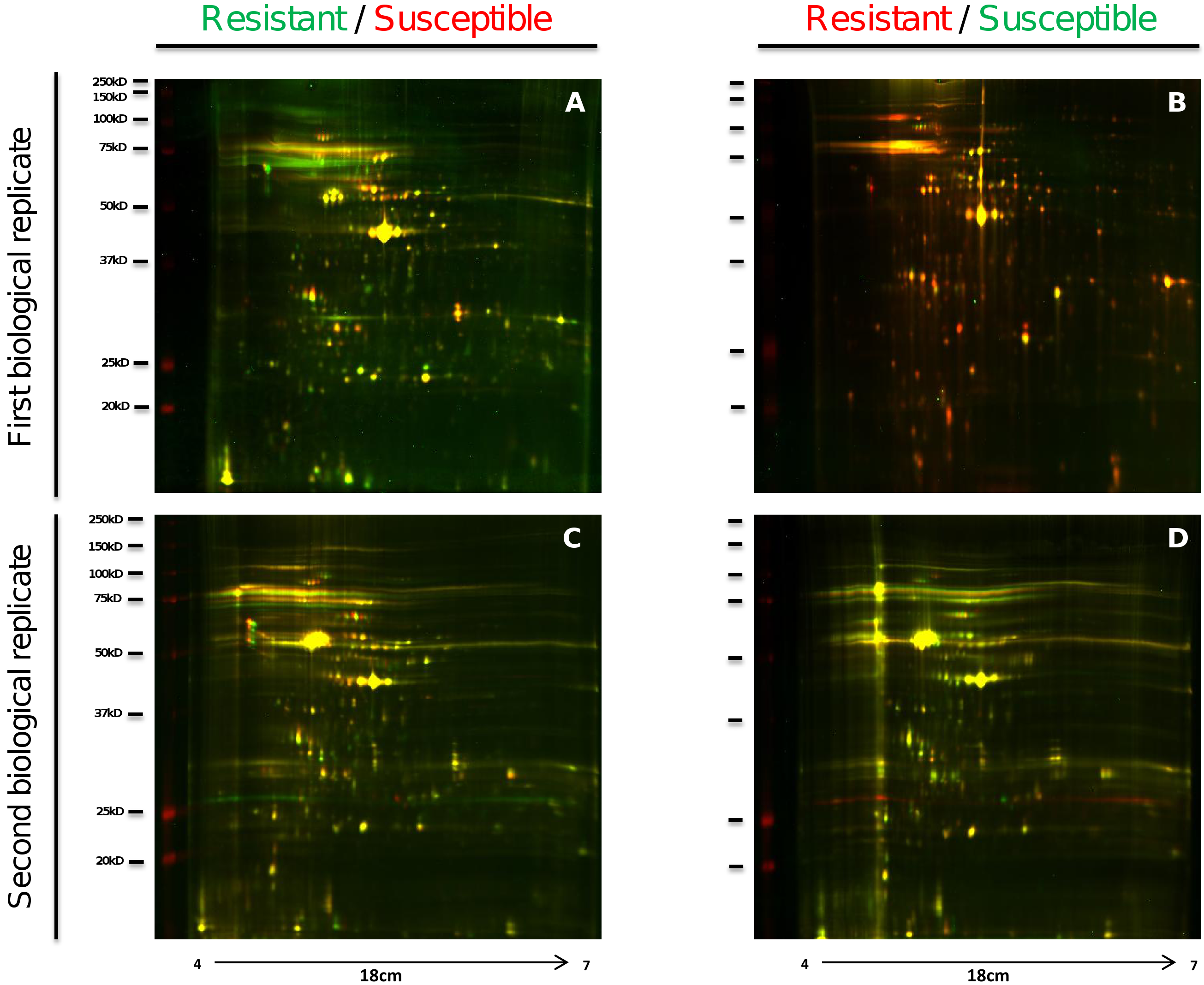

Supplement: Additional file 4 — 2D-DIGE gels from the two biological replicates and dye-swapping. BBMV prepared from first (A and B) and second (C and D) biological replicate are separated in function of their size (kDa) and their isoelectric point (pI). BBMV from Bti resistant strain are labeled with Cy3 and susceptible strain with Cy5 (A and C) or resistant strain with Cy5 and susceptible with Cy3 (B and D). [file 1471-2164-13-248-S4.tiff]

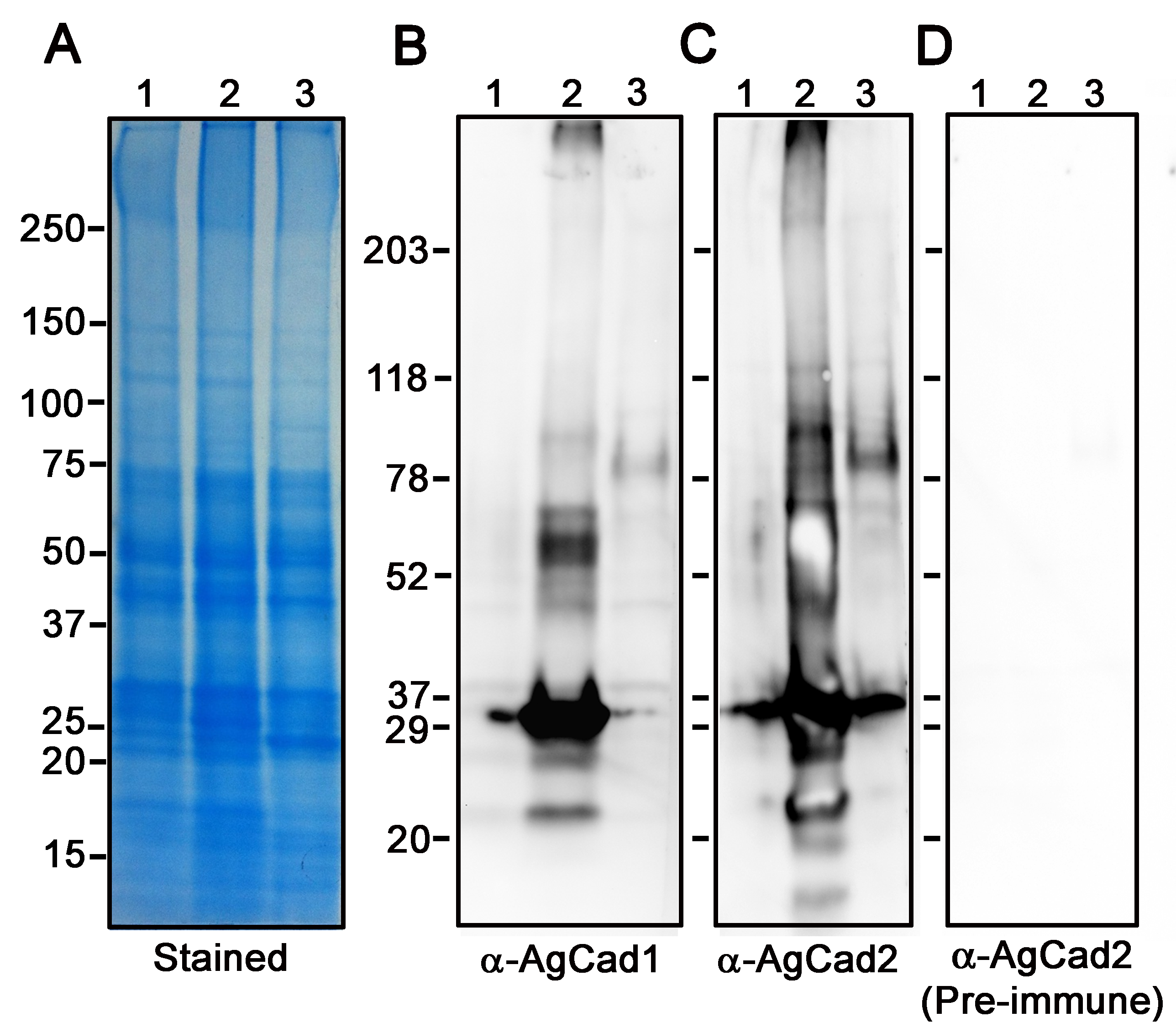

Supplement: Additional file 7 — Cadherin detection by immunoblotting. BBMV proteins from the susceptible Bora-Bora strain (lane 1), LiTOX strain (lane 2) and the UGAL Aedes strain (lane 3) were separated in SDS-PAGE and stained with coomassie blue (panel A) or probed with α-AgCad1 antibodies (panel B), α-AgCad2 antibodies (panel C) or with pre-immune serum from α-AgCad2 rabbit (panel D). [file 1471-2164-13-248-S7.tiff]
